# A Validation Study of Heart Rate Variability Index in Monitoring Basketball Training Load

**DOI:** 10.3389/fphys.2022.881927

**Published:** 2022-04-25

**Authors:** Naijing Jin, Jie Tian, Yue Li, Jing Mi

**Affiliations:** ^1^ Sports Coaching College, Beijing Sport University, Beijing, China; ^2^ Sports Department, Shenzhen Institute of Information Technology, Shenzhen, China

**Keywords:** heart rate variability, TL_HRV_, basketball, training load, quantification

## Abstract

This study aimed to investigate whether the heart rate variability index (TL_HRV_) during five ball-drills could be used to quantify training load (TL) in collegiate basketball players. Ten elite male college basketball athletes (18.2 ± 0.4 years) were recruited to perform five ball-drills (1V1, 2V2, 3V3, 4V4, and 5V5) which lasted 10 min and varied in intensity. During each drill, TL_HRV_, training impulse (TRIMP), rating of perceived exertion (RPE), speed, and distance were recorded by Firstbeat, Foster’s RPE scale, and SiMi Scout. The correlation (Spearman’s and Pearson’s correlation coefficient), reliability (intra-class correlation coefficient, ICC), and agreement (Bland-Altman plots) among TL_HRV_, TRIMP, RPE, speed, and distance were examined. TL_HRV_ was significantly correlated with TRIMP (r = 0.34, *p* = 0.015) and RPE (r = 0.42, *p* = 0.002). TL_HRV_ was significantly correlated with training intensity (r = 0.477, *p* = 0.006) but not with volume (r = 0.272, *p* = 0.056). TL_HRV_ and TRIMP, RPE showed significant intraclass relationships (ICC = 0.592, *p* = 0.0003). Moreover, TL_HRV_ differentiated basketball drills of equal volume and varying intensity. We concluded that TL_HRV_may serve as an objective and rational measure to monitor TL in basketball players.

## Introduction

A precise and reliable quantification of training load (TL) for each individual is given great importance in sports training. It could contribute to a more accurate assessment of how an athlete is responding to a prescribed training and assist in subsequent changes to the training program to avoid overtraining and improve training efficiency. The evaluation of TL varies between sports and training modalities, especially in basketball, football, and other team sports, which demand a hybrid energy supply, and which involve frequent physical contact, complex technical actions, varied tactics, and diverse training methods, making precise TL difficult to assess.

Currently, there are multiple methods of TL quantification in basketball. The external load measures include distance, speed, time-motion analysis, accelerometer load, and global positioning system (GPS) parameters, while measures such as heart rate (HR), blood lactate, and ratings of perceived exertion (RPE) are commonly used to assess internal load (Piedra et al., 2021). However, different TL evaluation tools and indicators have their advantages and limitations. For example, GPS devices provide a real-time measure of an athletes position, speed, and distance which is widely used in outdoor team sports, but it is susceptible to a variety of interferences owing to the low signal power, and thus presents poor measurement reliability during short high-speed movements and multidirectional exercises (Crang et al., 2021). Video techniques not only provide duration, distance, and speed during exercise but also accurate analysis of complex sport-specific technical actions (Carling et al., 2008). However, it is incapable of real-time measurements and is time-consuming to process. Traditional biochemical markers such as blood lactate concentration can accurately reflect the body’s response to exercise stimuli, but measurement precision is subject to errors derived from the blood sampling site, time, and technique. HR, as a non-invasive and convenient method, is commonly used for monitoring exercise intensity in the field, but studies have shown that HR and intensity of aerobic exercise are linearly correlated, while in interval and resistance training, the relationship between these two indicators is not linear (Borresen and Lambert, 2009). Thus, monitoring basketball training and competition load with HR alone may underestimate exercise intensity (Narazaki et al., 2009). RPE has been developed and validated as a simple and effective method to assess the internal TL of athletes in different sports. However, athletes may exaggerate their RPE in some situations, considering that subjective sensations are influenced by many factors, including physical health, energy supply, and psychological state (Fox et al., 2017). In addition, the RPE value may be different for different training purposes. For example, Lupo et al. found that the intensity of taekwondo competition determined by RPE which tends to be lower than that measured by HR-based methods. Conversely, RPE was more consistent with HR-measured TL in training sessions (Lupo et al., 2017). The training impulse (TRIMP) is a measure that reflects the comprehensive response of the body to internal and external load stimuli through training volume (duration) and intensity (average HR). However, some studies have shown that TRIMP cannot distinguish between continuous and interval training of the same duration and average HR, which limits its application in short-duration, high-intensity interval training (Buchheit and Laursen, 2013). Therefore, in practice, there is no real gold standard for TL quantification.

Recently, heart rate variability (HRV) has been widely used to monitor training as a non-invasive quantitative assessment of cardiac autonomic tone and balance, which is sensitive to changes of psychophysiology and fatigue of athletes under external stimulation (Deus et al., 2019; Schneider et al., 2019; Lukonaitiene et al., 2020). The root mean square of successive differences between beat intervals (RMSSD), as a time-domain marker of HRV, is very sensitive to the short-term and rapid regulation of vagus nerve activity in the autonomic nervous system (Rave et al., 2020). During exercise, the release of norepinephrine triggers sympathetic activation, which leads to increased cardiac contractility and conduction of electrical signals to meet the physiological needs of exercise. At the same time, the vagus nerve is inhibited and RMSSD declines dramatically. When at rest, the parasympathetic vagus nerve promotes recovery by releasing acetylcholine, slowing down the HR, reducing cardiac contractility, and inhibiting cardiac electrical signaling speed, and thus sympathetic nerve activity is inhibited, and RMSSD increases significantly. RMSSD declines sharply during exercise and then becomes reactivated after exercise, which can accurately reflect the stress response of different individuals to TL (Shaffer et al., 2014; Gordan et al., 2015; Grassler et al., 2021). Saboul et al. proposed the HRV index (TL_HRV_) as a new TL quantification tool based on RMSSD related research and verified the effectiveness of TL_HRV_ in TL assessment of continuous and interval running (Saboul et al., 2016). Zhao et al. further demonstrated that TL_HRV_ was in line with TRIMP in evaluating the TL of continuous exercise (Zhao et al., 2018). However, there is still uncertainty whether TL_HRV_ could effectively quantify TL in high-intensity interval events such as basketball and football.

Thus, the purpose of this investigation was to verify whether TL_HRV_ in assessing the TL of basketball is in line with TRIMP, RPE, speed, and distance during five ball-drills (half-court 1V1, 2V2, 3V3, 4V4, and 5V5) or not. It was hypothesized that TL_HRV_ was able to differentiate loads between the five basketball drills, and it showed a good agreement and correlation with the validated internal and external load monitoring indicators.

## Materials and Methods

### Participants

Based on the previous literatures (Castagna et al., 2011; Torres-Ronda et al., 2016; Saboul et al., 2016; Zhao et al., 2018; González-Fimbres et al., 2020), it was assumed that different intensities of ball-drills had a strong effect on HRV, and a sample size calculator (G∗Power 3.1, Germany) was used to calculate the sample size required by this study. Specifically, an effect size (f) was set to 0.35, an *α*-error probability was set to 0.05, and the power was 0.08. The final calculation results suggested that a total of about N = 10 persons would be required. Accordingly, ten elite collegiate male basketball players (competition level: national; training years = 7.8 ± 1.2 years; age = 18.2 ± 0.4 years; height = 185.8 ± 6.8 cm; body mass = 80.2 ± 6.7 kg) were invited to take part in the study. Before the experiment, the physical health of the subjects was investigated using medical questionnaires to ensure that all the subjects were free from any injury and disease. The study followed all the principles of the Helsinki Declaration and was approved by the Academic Ethics Committee of the Beijing Sport University.

### Experimental Design

This observational within-subjects study lasted 2 weeks. All 10 athletes participated in five half-court ball-drills adopted some of the game rules for three-man basketball: one-a-side (1V1), two-a-side (2V2), three-a-side (3V3), four-a-side (4V4), and five-a-side (5V5). The five ball-drills were classified according to the confrontation format in terms of the number of players, and the confrontation intensity increases with a decrease in the number of athletes on the court. These ball-drills were chosen because they are commonly reported to be a classic and effective practice for inducing specific physical and skill adaptations to basketball players. Each drill lasted 10 min and varied in intensity (Castagna et al., 2011). To avoid the influence of biorhythms on test results, each drill was performed in random order and at the same time each day, at 2-day intervals. All the subjects were required to refrain from any specific basketball training other than ball-drills and self-selected strength and conditioning programs during the test period, and avoid the intake of alcohol and coffee beverages prior to each test.

The 10 basketball players were divided into groups A and B according to their competitive level and position, with two guards, two forwards, and one center in each group. Each player in group A was marked as guard A1, guard A2, forward A3, forward A4, and center A5, and group B was numbered in the same way. Additionally, two guard substitutes in 2V2 (marked as S1 and S2), two center substitutes in 3V3 (marked as S3 and S4), and two forward substitutes (marked as S5 and S6), two guard substitutes (S1 and S2), and two center substitutes (S3 and S4) in 4V4 were added in order to ensure the same number of athletes in each group. The test data of the substitutes were not included in the final analysis. Each athlete was asked to try their best to play against each other under the supervision and guidance of two professional coaches on the court.

The specific grouping was as follows:

Group A: A1 YU; A2 WANG; A3 YU; A4 WANG; A5 WAN
Group B: B1 ZHANG; B2 GAO; B3 YE; B4 XIE; B5 TIAN

The specific test matchups were as follows:

1) 1V1.

A1 vs. B1; A2 vs. B2; A3 vs. B3; A4 vs. B4; A5 vs. B5

2) 2V2.

A1, A5 vs. B1, B5; A2, A3 vs. B2, B3; A4, S1 vs. B4, S2

3) 3V3.

A1, A3, A5 vs. B1, B3, B5; A2, A4, S3 vs. B2, B4, S4

4) 4V4.

A1, A3, A4, A5 vs. B1, B3, B4, B5; A2, B2, S3, S5 vs. S1, S2, S4,S6

5) 5V5.

A1, A2, A3, A4, A5 vs. B1, B2, B3, B4, B5

### Measures

Internal load measures included TL_HRV_, TRIMP, and RPE; while external load measures included running distance and speed.

1) TL_HRV_: The TL_HRV_ was calculated based on the method defined by Saboul et al. (2016). The specific formula is shown in Eq. 1:

$$TL_{HRV}=\ln\left(\text{T×}\frac{\text{Pre}5-\text{Post}5}{\text{Post}30-\text{Post}5}\right)$$ (1)

where TL_HRV_: heart rate variability index.T: duration of the drill.Pre5: RMSSD 5 min before training.post5: RMSSD 5 min after training.Post30: RMSSD 30 min after training.

The TL_HRV_ formula mainly consisted of three different 5-min RMSSDs: pre5 RMSSD (RMSSD for 5 min in a quiet state before warming up for each test); post5 RMSSD (RMSSD in the 5th to 10th min of the recovery phase after each test), post30 RMSSD (RMSSD in the 30th to 35th min of the recovery phase after each test), which was collected using Firstbeat SPORTS equipment. The first part of the formula (pre5-post5) accounted for the disturbance of exercise to the body, and the change of RMSSD before and after exercise reflected the body’s response to different intensities of exercise. The second part (post5-post30) reflected the homeostatic process of the body from intense response to rest state after exercise cessation. The effect of exercise on vagus nerve reactivation was assessed by the increase of RMSSD after exercise. The ratio between the two parts of the formula normalized changes in HRV, reducing the effects of sleep, diet, and stress on daily baseline HRV fluctuations.

The RMSSD indicator was chosen as the main indicator of the formula for the following reasons: 1) RMSSD represents changes in HRV over a short period time, particularly the role of the vagus nerve in regulating body functions; 2) RMSSD is one of the most commonly used time-domain parameters in HRV, and it is less affected by respiratory rate and measurement of posture (which can be used for non-supine lying) and has higher reliability and maneuverability than the frequency domain parameters.

2) TRIMP: The HR and TRIMP of athletes during exercise was recorded by the Firstbeat SPORTS equipment. TRIMP was calculated according to Banister’s method (Lucia et al., 2003), as shown in Eq. 2.

$$\text{TRIMP}=\text{T}\times \frac{{\text{HR}}_{\text{exe}}-{\text{HR}}_{\text{rest}}}{{\text{HR}}_{\text{max}}-{\text{HR}}_{\text{rest}}}\times 0.64\times {\text{e}}^{1.92\times \frac{{\text{HR}}_{\text{exe}}-{\text{HR}}_{\text{rest}}}{{\text{HR}}_{\text{max}}-{\text{HR}}_{\text{rest}}}}$$ (2)

where TRIMP: training impulse.T: duration of the drill.HR_exe_: mean heart rate during the drill.HR_rest_: basal heart rate.HR_max_: maximum heart rate.e: natural logarithm, valued at 2.712.

3) RPE: The RPE of each athlete was recorded 30 min after each training session, using Foster’s RPE scale (0–10) (Foster et al., 1995).
4) Running speed and distance: Each test was videotaped live using a professional digital camera (Sony NX100). The video was then imported into the SIMI Scout software to calculate the distance and speed of the players in each drill. Before the analysis, the coordinates of four points were established for the field in the video to form a two-dimensional coordinate system, and the values of the four coordinate points were determined according to the length and width of the basketball court. The coordinates of the four points were (0, 0), (0, 1,400), (1,500, 1,400), and (1,500, 0), respectively, based on the point where the bottom line and the left edge line intersected as the origin.

### Statistical Analyses

All the data were processed by SPSS statistical software 21.0 (SPSS Inc., Chicago, IL, United States ). The data were shown as mean ± SD, and the significance was set at *p* < 0.05. The Shapiro-Wilk test was used to test the normality of the data. If the data were normally distributed, one-way repeated measures analysis of variance was used to compare the differences between different drills, the LSD method was used for post-test, and the Pearson’s correlation coefficient was used for correlation analysis. . If not, the Friedman test was used for between-group difference analysis, the Wilcoxon signed rank test was used for post hoc analysis, and the Spearman’s correlation coefficient was used for correlation analysis.The agreement among TL_HRV_, TRIMP, and RPE was examined by the Bland-Altman test and intraclass correlation coefficient. Since the three measurements have different units, the percentage conversion method defined by Saboul et al. was used to convert the values measured for each indicator into a percentage; for example, TRIMP_1V1_ = 100×[TRIMP_1V1_/(TRIMP_1V1_+TRIMP_2V2_+TRIMP_3V3_+TRIMP_4V4_+TRIMP_5V5_)] (Saboul et al., 2016).

## Results

### The Normality of the Data

The normality of the distribution of the five measurements is shown in Table1. All the variables showed a normal distribution except RPE (*p* = 0.001) and speed (*p* = 0.000). Therefore, the Friedman test was used for between-group difference analysis of RPE and speed, one-way repeated measures analysis of variance was used to compare the differences of TL_HRV_, TRIMP, and distance. Otherwise, the correlation among TL_HRV_, speed, and RPE was examined by Spearman’s correlation coefficient, while the correlation among TL_HRV_, TRIMP, and distance was examined by Pearson’s correlation coefficient.

**TABLE 1:** The normality of the distribution of the five measurements.

Measures	Mean ± SD	Statistic	Df	Sig
TL_HRV_	2.99 ± 0.5	0.971	50	0.262
RPE	6.12 ± 1.14	0.910	50	0.001
TRIMP	22.4 ± 5.35	0.961	50	0.098
Distance	797.39 ± 92.83	0.972	50	0.269
Speed	3.54 ± 0.73	0.678	50	0.000

### Assessment of Internal and External Load

The TL of each drill was assessed by TRIMP, TL_HRV_, and RPE. Results are shown in Table 2.

**TABLE 2:** Internal load assessment of five ball-drills.

Training Session (V)	TRIMP	TL_HRV_	RPE
1V1	24.8 ± 4.5	3.7 ± 0.4	7.0 ± 1.2
2V2	24.8 ± 4.6	3.2 ± 0.5^a^	6.6 ± 0.7
3V3	23.3 ± 4.7	2.8 ± 0.6^ab^	6.3 ± 0.8
4V4	20.1 ± 4.8^abc^	2.7 ± 0.4^ab^	5.3 ± 0.5^abc^
5V5	19 ± 4.9^abc^	2.5 ± 0.8^abc^	5.4 ± 1.2^ab^

a p < 0.05: different from 1V1.

^b^p < 0.05: different from 2V2.

^c^p < 0.05: different from 3V3.

^d^ *p* < 0.05: different from 4V4.

TRIMP showed significant TL differences among the five drills (*p* = 0.004). Specifically, there was a TL difference between 1V1 and 4V4 (*p* = 0.001); 1V1 and 5V5 (*p* = 0.015); 2V2 and 4V4 (*p* = 0.002); 2V2 and 5V5 (*p* = 0.002); 3V3 and 4V4 (*p* = 0.015); 3V3 and 5V5 (*p* = 0.017), but not between 1V1 and 2V2 (*p* = 1.0); 1V1 and 3V3 (*p* = 0.251); 2V2 and 3V3 (*p* = 0.081); 4V4 and 5V5 (*p* = 0.577). TL_HRV_ showed significant TL differences among the five drills (*p* = 0.004). Specifically, there was a TL difference between 1V1 and 2V2 (*p* = 0.007); 1V1 and 3V3 (*p* = 0.0001); 1V1 and 4V4 (*p* = 0.00005); 1V1 and 5V5 (*p* = 0.0001); 2V2 and 3V3 (*p* = 0.002); 2V2 and 4V4 (*p* = 0.0003); 2V2 and 5V5 (*p* = 0.0004); 3V3 and 5V5 (*p* = 0.015), but not between 3V3 and 4V4 (*p* = 0.053); 4V4 and 5V5 (*p* = 0.057). Finally, RPE showed significant TL differences among the five drills (*p* = 0.001). Specifically, there was a TL difference between 1V1 and 4V4 (*p* = 0.002); 1V1 and 5V5 (*p* = 0.011); 2V2 and 4V4 (*p* = 0.006); 2V2 and 5V5 (*p* = 0.024); 3V3 and 4V4 (*p* = 0.028), but not between 1V1 and 2V2 (*p* = 0.777); 1V1 and 3V3 (*p* = 0.396); 2V2 and 3V3 (*p* = 0.572); 3V3 and 5V5 (*p* = 0.090); 4V4 and 5V5 (*p* = 0.621).

Data related to running distance are shown in Table 3. We found significant TL differences among the five drills (*p* = 0.000001). Specifically, there was a TL difference between 1V1 and 5V5 (*p* = 0.0003); 2V2 and 4V4 (*p* = 0.002); 2V2 and 5V5 (*p* = 0.0005); 3V3 and 4V4 (*p* = 0.0002); 3V3 and 5V5 (*p* = 0.00008), but not between 1V1 and 2V2 (*p* = 0.164); 1V1 and 3V3 (*p* = 0.065); 1V1 and 4V4 (*p* = 0.107); 2V2 and 3V3 (*p* = 0.532); 4V4 and 5V5 (*p* = 0.106).

**TABLE 3:** The running distance and speed of five ball-drills.

Training Session (V)	Distance (m)	Mean Speed (m/s)
1V1	795.6 ± 85.2	3.9 ± 0.1
2V2	846.9 ± 57.9	4.0 ± 0.1
3V3	858.9 ± 87.4	4.0 ± 0.1
4V4	728.3 ± 84.2^bc^	3.3 ± 0.8^b^
5V5	690.6 ± 62.5^abc^	2.8 ± 0.4^abc^

^a^p < 0.05: different from 1V1.

^b^p < 0.05: different from 2V2.

^c^p < 0.05: different from 3V3.

^d^ *p* < 0.05: different from 4V4.

The speed of the five drills is presented in Table 3. We found significant TL differences among the five drills (*p* = 0.000). Specifically, there was a TL difference between 1V1 and 5V5 (*p* = 0.015); 2V2 and 4V4 (*p* = 0.015); 2V2 and 5V5 (*p* = 0.000); 3V3 and 5V5 (*p* = 0.019), but not between 1V1 and 2V2 (*p* = 1); 1V1 and 3V3 (*p* = 1); 1V1 and 4V4 (*p* = 0.562); 2V2 and 3V3 (*p* = 1); 3V3 and 4V4 (*p* = 0.66); 4V4 and 5V5 (*p* = 1).

### Correlations Between Internal and External Measures of Load and TL_HRV_

As shown in Table 4, Spearman’s correlation coefficient showed moderate associations between TL_HRV_ and TRIMP (r = 0.34, *p* = 0.015), as well as large associations between TL_HRV_ and RPE (r = 0.42, *p* = 0.002). Correlations between TL_HRV_ and external load measures (Table 5) showed significant correlations between TL_HRV_ and speed (r = 0.447, *p* = 0.006), but no correlations between TL_HRV_ and distance (r = 0.272, *p* = 0.056).

**TABLE 4:** Correlation between TL_HRV_ and internal measured variables.

	TL_HRV_	TRIMP	RPE
TRIMP	0.34^*^	1	0.47^**^
RPE	0.42^**^	0.47^**^	1
TL_HRV_	1	0.34^*^	0.42^**^

*p < 0.05 was considered significant and **p < 0.01 highly significant.

**TABLE 5:** Correlations between TL_HRV_ and external load measures.

	Distance	Speed
TL_HRV_	0.272	0.477^**^

**p < 0.01 was considered highly significant.

**FIGURE 1:**
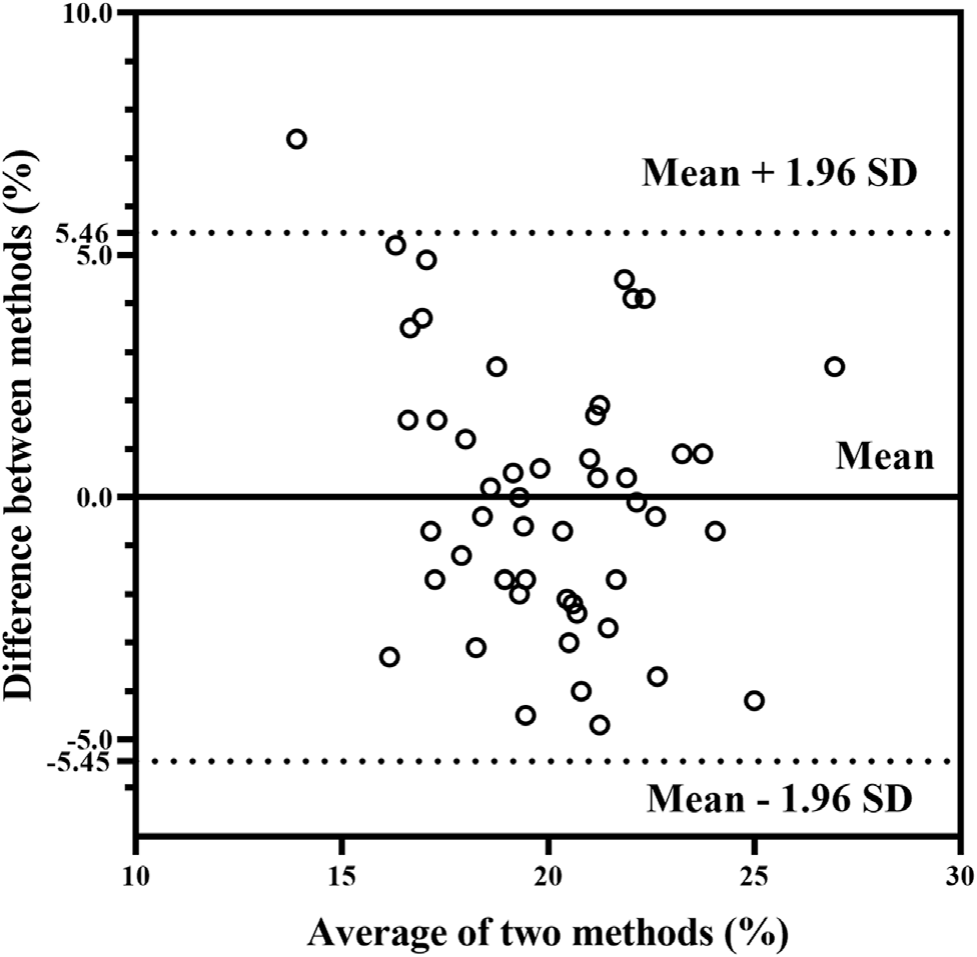
Bland and Altman plots between TL_HRV_ and TRIMP.

**FIGURE 2:**
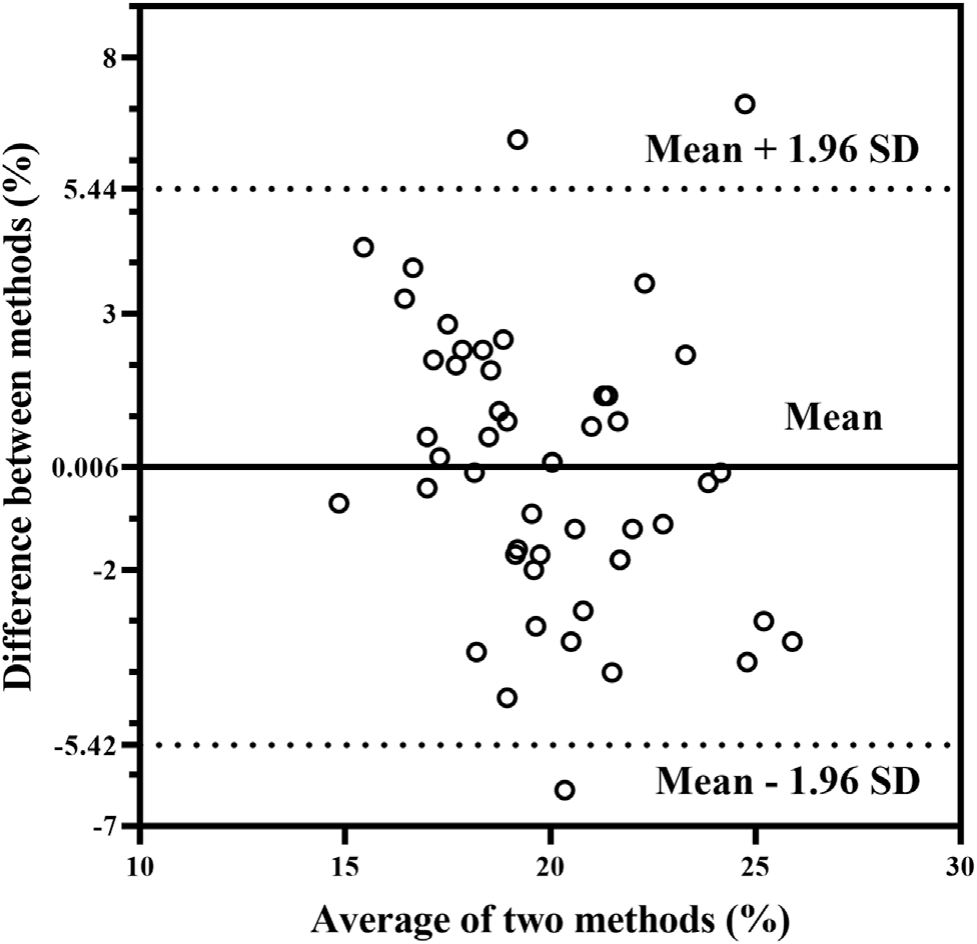
Bland and Altman plots between TL_HRV_ and RPE.

The agreement among TL_HRV_, RPE, and TRIMP presented in Figure 1 and Figure 2 shows that the *x*-axis and *y*-axis values are normally distributed, and the mean deviation of the percentage conversion values of the two indicators were 0.006 (solid black line in Figure 1 and Figure 2), which are both close to zero. Moreover, the percentage conversion values fall mostly between the 95% limits of agreement (mean ±1.96 SD), with only one point for TRIMP; three points for RPE are not included (less than 5%). According to the intraclass correlation coefficient results (Table 6), there was a positive relationship between TL_HRV_, RPE, and TRIMP (r = 0.592, *p* = 0.0003).

**TABLE 6:** The intraclass correlation coefficients between TL_HRV_, RPE, and TRIMP.

	r	95% CI		F			
		Lower limit	Upper limit	Value	df1	df2	Sig
Single measurement	0.592	0.439	0.725	5.281	49	98	0.0003
Average measurement	0.813	0.701	0.888	5.281	49	98	0.00002

## Discussion

The purpose of this investigation was to verify the effectiveness of TL_HRV_ in quantifying the TL of basketball, by exploring the relationship between TL_HRV_ and internal and external load measures during five ball-drills in trained athletes. It should be noted that this is the first study to evaluate the TL of basketball by TL_HRV_. The current study found that TL_HRV_ was able to differentiate loads between various basketball drills, and it showed good agreement and correlation with TRIMP, RPE, and running speed.

The results showed that TL_HRV_ of 1V1, 2V2, 3V3, 4V4, and 5V5 decreased gradually with the increase of the number of athletes in each group. This may be attributed to the fact that the number of players affected the physical demands of basketball, which stimulated the parasympathetic nerves to innervate the arteries and myocardium by releasing acetylcholine to produce corresponding adaptive physiological changes. This result is in line with those of previous observations. For example, Castagna et al. found that the fewer players on the court, the higher the average heart rate and blood lactate concentration of athletes during 5V5, 3V3, and 2V2 drills (Castagna et al., 2011). The percentage of maximal HR and peak VO_2_ has been reported to increase by 3%–8% and 10%, respectively, with a decrease in the number of players in basketball small sided games (Conte et al., 2016). In fact, the number of athletes on the court causes differences in factors such as the frequency of techniques, the rhythm of the game, and the relative court area per player (Fernandez-Espinola et al., 2020). Specifically, the fewer players on the court, the more opportunities each player has to touch the ball, and it can increase the individual physical contacts, technical actions, and the relative court area per player, thereby changing the physical requirements of the exercise, leading to the differences in TL of the five drills. Conversely, Wang et al. showed that the TL of 4V4 and 3V3 were higher than 5V5, 2V2, and 1V1 in a half-court game (Wang et al., 2018). This may be related to the different amount of work each group was carrying out in their experiment. The training durations of 1V1, 2V2, 3V3, 4V4, and 5V5 were 9, 11, 9, 13, and 16 min, respectively. Comparing the intensity of different forms of exercise under different training durations may cause some deviation in research results.

The results of the analysis clearly demonstrated the correlation between the two internal TL variables and TL_HRV_; similar results were found in previous research related to various indicators of HRV. Zhao et al. have investigated correlations between TL_HRV_ and TRIMP in female soccer athletes; they investigated TL_HRV_ at 60% and 85% maximal speed of the graded maximal test and found moderate correlations between TL_HRV_ and TRIMP (Zhao et al., 2018). Moderate negative correlations between post5 RMSSD and TRIMP were reported by González-Fimbres in the high-intensity interval and low-intensity continuous running of youth triathletes (González-Fimbres et al., 2020). Orellana et al. observed moderate correlations between post10 RMSSD and TRIMP by examining 14 healthy men who ran at ventilatory thresholds (VT1 and VT2) and maximal aerobic speed (Orellana et al., 2019). As for the relationship of HRV and RPE, moderate correlations between morning HRV and session ratings of perceived exertion (sRPE) of the previous day were found by Sartor et al. in young male elite gymnasts (Sartor et al., 2013). Kaikkonen et al. illustrated larger negative correlations between RPE and HF, and TP in interval running exercises with different intensities and durations (Kaikkonen et al., 2012). The studies above suggest that HRV is related to TRIMP and RPE in various sports and exercise, a possible explanation for this might be that the three indicators are based on the relationship between HR and exercise intensity, which can reflect the change of cardiac function with TL during exercise.

In the present study, a significant relationship was found between TL_HRV_ and speed. Interestingly, the distance was not related to TL_HRV_. Similar conclusions were reported by Seiler et al., who explored the effect of exercise duration on RMSSD in endurance athletes, finding that post-exercise RMSSD was not affected by duration, despite a two-fold increase in duration (Seiler et al., 2007). Research by Kaikkonen et al. showed that HRV recovery was slower with increased exercise intensity, while doubling distance had no effect on HRV recovery (Kaikkonen et al., 2007). Saboul et al. also did not find a correlation between TL_HRV_ and exercise volume in long-distance male runners and argued that regardless of the type of exercise, exercise intensity was the main factor affecting baseline HRV and recovery of the body post-exercise (Saboul et al., 2016). Based on the above evidence, we believe that high-intensity training may delay HRV recovery, and the higher the exercise intensity, the lower the RMSSD in the same recovery time. Moreover, it is also suggested that high-intensity exercise (above the second ventilatory threshold) seems to have a greater effect on parasympathetic regulation of cardiac activity compared with exercise at very low intensity (below the first ventilatory threshold) (Kaikkonen et al., 2010). This is partly explained by the fact that low-intensity exercise mainly relies on the aerobic energy system, when blood lactate concentrations generally do not exceed the resting value. In contrast, high-intensity exercise is primarily powered by the anaerobic glycolytic metabolic system, which leads to a large increase in blood lactate concentrations during exercise, triggering metabolic reflexes that inhibit parasympathetic activity, and in turn lower HRV. Collectively, these physiological mechanisms create intuitive rationales for the significant associations between running speed and TL_HRV_ found in our analyses (González-Fimbres et al., 2020).

The aim of sports training is to disturb the body’s homeostasis by applying appropriate stimuli to the body, including sympathetic and vagal activity. Monitoring whether such stimulation is suitable for the body using precise and convenient methods or tools is a milestone in athletic training. Although there is no “gold standard” to quantify TL, TRIMP may serve as a reference parameter that partially reflects the body’s physiological response to stress. In addition, some studies found that RPE was correlated with the cardiorespiratory and metabolic demands of the body during exercise, and therefore had been also used as a reference index for TL evaluation. However, the measurement of TRIMP cannot distinguish between continuous and interval training of the same duration and average HR, which limits its application in short-duration, high-intensity interval training (Buchheit and Laursen, 2013). RPE is simple and effective in training practice, but it is subjective and cannot directly reflect the athletes’ physiological response during training (Kaikkonen et al., 2012). In pursuit of an objective and convenient physiological indicator to quantify TL in basketball, this study investigated the emerging TL_HRV_ parameter. The results of the study were basically in line with the expected hypothesis, that is, the ball-drills of different intensity did show some differences under the quantification of TL_HRV_. Accordingly, we cautiously conclude that TL_HRV_ could be used as markers of training load and psychophysiological status of male basketball players, as it can provide unique and deep insights into athletes’physiology response compared to TRIMP, RPE, speed, and distance. Furthermore, it is easy for practitioners to obtain the RMSSD indicators and the relevant data analysis for free from some HR monitoring systems. From a practical point of view, we encourage practitioners to monitor changes in TL_HRV_ during a training session, and may even attempt to monitor it continuously over a training period, which could contribute to a more objective and accurate assessment of how an athlete is responding to a prescribed training and assist in subsequent changes to the training program to avoid overtraining and improve training efficiency.

In this study, the correlation analysis between TL_HRV_ and internal load indicators (TRIMP and RPE) and external load indicators (running distance and speed) was used to preliminarily verify that TL_HRV_ can be used to evaluate TL in basketball. However, the study was limited by the absence of analysis concerning biochemical indicators such as blood lactate concentration. Therefore, in future research, it would be beneficial to correlate TL_HRV_ with more physiological and biochemical indicators to further confirm its accuracy in basketball TL evaluation.

Furthermore, the TL_HRV_ formula is based on pre5 RMSSD, post5 RMSSD, and post30 RMSSD. We should acknowledge that it takes a certain amount of time to collect RMSSD data at three time points, which is more complicated than collecting RPE data for daily use. In addition, this formula has not been extensively tested across different sports and training modalities, and its applicability to load monitoring in other forms of exercise is still unclear. From this perspective, future research could focus on demonstrating the validity of TL_HRV_ in different sports and training modalities.

## Conclusion

Based on the present study, we cautiously conclude that TL_HRV_ may serve as an objective and rational measure for monitoring TL in basketball players. In basketball training practice, it is suggested that coaches can judge the physiological adaptation of athletes to training load according to changes in TL_HRV_ after training sessions, which will help practitioners to adjust training plans and meet specific training goals.

## Data Availability

The original contributions presented in the study are included in the article/Supplementary Material, further inquiries can be directed to the corresponding author.
